# Concerning immune synapses: a spatiotemporal timeline

**DOI:** 10.12688/f1000research.7796.1

**Published:** 2016-03-31

**Authors:** Alvaro Ortega-Carrion, Miguel Vicente-Manzanares

**Affiliations:** 1 Immunology Section, Department of Medicine, Universidad Autonoma de Madrid School of Medicine, Madrid, Spain

**Keywords:** Immune synapse, T-Cell, antigen presenting cell, T cell activation

## Abstract

The term “immune synapse” was originally coined to highlight the similarities between the synaptic contacts between neurons in the central nervous system and the cognate, antigen-dependent interactions between T cells and antigen-presenting cells. Here, instead of offering a comprehensive molecular catalogue of molecules involved in the establishment, stabilization, function, and resolution of the immune synapse, we follow a spatiotemporal timeline that begins at the initiation of exploratory contacts between the T cell and the antigen-presenting cell and ends with the termination of the contact. We focus on specific aspects that distinguish synapses established by cytotoxic and T helper cells as well as unresolved issues and controversies regarding the formation of this intercellular structure.

## Introduction

The immune synapse (IS) is a central event in the development of the adaptive immune response that results in the activation of the T cell. The “synapse-like” nature of the intimate contact between the T cell and the antigen-presenting cell (APC) during T cell activation was initially proposed by Norcross in the early 1980s ^1^, although the term “immunological synapse” first appeared in a review by Paul and Seder in 1994 ^2^. The specifics of molecular segregation into activation clusters at the T cell:APC interface dates back to the seminal observations of Kupfer’s group in 1998 ^3^. At the same time, Dustin and Shaw conjoined both concepts (the IS as the physical manifestation of T cell activation, and molecular segregation as the functional reflection of the T cell:APC interaction), adding crucial early data on the composition of the activation clusters ^4^. The IS can be defined as a stimulus-driven, spatiotemporal segregation of molecules that participate in T cell activation. Segregation requires the establishment of an intimate contact between a T lymphocyte and an APC. The molecular redistribution is antigen dependent, requiring the interaction of an antigen-specific T cell receptor (TCR) with an antigen-loaded major histocompatibility complex (MHC) molecule. The features and outcome of the IS depend on the type of T cell and APC. The interaction of a CD4+ T helper (T _H_) cell with an antigen-loaded MHC-II-bearing APC results in the specific recognition of the antigen and the activation of the T cell, i.e. proliferation, cytokine secretion, expression of effector molecules, etc. In the case of CD8+ T (CTL) cells interacting with cells displaying antigen-associated MHC-I, the outcome depends on the pre-exposure of the CTL to the antigen. Naïve CTL encountering specific antigens presented by APCs (e.g. dendritic cells [DCs] expressing antigen associated with class I via cross-presentation) are primed (“armed”) to kill target cells and proliferate. Primed CTL also form transient IS with target cells (tumor cells or cells infected by a virus), resulting in specific killing.

The IS displays remarkable similarities with the neuronal synapse (NS), to which it owes its name. For spatial and functional reference, the APC is better compared to the pre-synaptic terminal, and the T cell to the post-synaptic terminal. The presynaptic portal provides the initiating signal, soluble in the NS (neurotransmitters), but membrane bound in the IS (antigen-bearing MHC). Upon ligation of the key receptor in the post-synaptic terminal (neurotransmitter receptors in the NS; TCR and its signaling co-receptor CD3 in the IS), downstream signaling ensues, including calcium mobilization, actin remodeling, and functional activation of the post-synaptic cell ^1, 5^. However, a unique feature of the IS consists of specific antigenic recognition, which is absent in the central nervous system (CNS). Another difference is the duration of the contact: whereas some NS can last for days, weeks, or even months, IS between CTL and target cells resolve in minutes, whereas between T _H_ cells and APCs they can last from several hours to two days ^6, 7^. This feature change implies a different meaning for the concept of plasticity. In the NS, it refers to the modifications to the post-synaptic terminal that involve the consolidation and adaptation of the post-synaptic terminal to the flux of signal stemming from the pre-synaptic portal. In the IS, plasticity follows contact resolution and could be used to describe the functional changes to the T cell caused by the establishment of a productive synapse. These include activation (T _H_), activation (naïve CTL) or kill (primed CTL), and functional anergy or apoptosis, e.g. during thymic selection of naïve T cells. A major manifestation of functional plasticity is the development of immunological memory, i.e. the generation of long-lived T cells primed to respond to a specific antigen that trigger a much faster and more efficient response to repeated exposure to the same antigen.

## Overview of the spatiotemporal events of the IS

The study of the IS has focused on the establishment of hierarchical, spatiotemporally segregated events during the contact between the APC and the T cell. These events include the following:

1) Establishment of low-affinity, exploratory contacts between the T cell and the APC
2) Initial, scattered contact of the TCR with the antigen-loaded MHC on the APC, followed by initiation of TCR-dependent signaling pathways upon specific recognition of the MHC-peptide complex. Such activation is “umbrella shaped” (simultaneous activation and amplification of multiple pathways through different sets of effectors) and induces the activation of multiple effectors, including membrane-bound molecules, e.g. integrins, signaling adaptors, cytoskeletal elements, and transcription factors
3) Transactivation of adhesion molecules (integrins) that consolidate the interaction between the T cell and the APC. This step actually begins after initial TCR activation (step 2), but they evolve in parallel
4) Cytoskeleton- and signaling-dependent clustering of adhesion molecules and the TCR/CD3 complex at the contact interface between the T cell and the APC. In most cases, clustering is spatiotemporally segregated, i.e. the TCR/CD3 clusters and the integrin clusters, and their respective sets of adaptors, are separated
5) Signaling- and motor-dependent positioning of the secretory apparatus (including microtubules and microtubule-binding proteins) to the contact interface of the T cell
6) (Primed CTL only, also natural killer [NK] cells) Actin clearance at the center of the contact interface, enabling a tight association of the secretory apparatus with the plasma membrane
7-i) (T _H_ cells) Stabilization of the contact and transcriptional activation of the T cell, including cytokine production and the expression of activation markers
7-ii) (Naïve CTL) Stabilization of the contact, priming and activation
7-iii) (Primed CTL and NK cells) Degranulation and target cell killing
8) Termination of the contact

From this flowchart, it becomes obvious that a major difference between the IS established by CTL and that established by T _H_ cells is the overall duration of the process and its immediate repeatability. CTL contacts are quick (to eliminate target cells rapidly), and CTLs can establish multiple IS with different target cells over short periods of time. Conversely, T _H_ cells establish prolonged IS and do not form consecutive IS once activated properly.

In the following sections, we will develop emerging concepts pertaining to each of these spatiotemporal events.

### Exploratory contacts

Exploratory contacts are mediated by low-affinity interactions between specific ligands and receptors. A major factor is the glycocalyx, which establishes charge-dependent repulsive interactions between the APC and the T cell (reviewed in 8). Additional contacts are mediated by glycosylation-dependent, low-affinity interactions, e.g. via galectins. For example, galectins bind TCR molecules with low affinity, thus the TCR does not activate ^9^. Antigen-loaded MHC molecules successfully compete with galectin to trigger TCR/CD3 activation and subsequent cytoskeletal remodeling and transcriptional activation (see below). Chemokine receptors also participate in the formation and subsequent stabilization of the initial contacts and localize in the IS. Possible functions for chemokine receptors in this subcellular region are likely to involve co-stimulation, cell attraction, enhancement of actin polymerization, etc. ^10^. Other exploratory contacts depend on specific protein-protein interactions, e.g. LFA-1 (α _L_β _2_) (APC) with ICAM-3 (T cell) ^11^, and LFA-3 (APC) with CD2 (T cell). LFA-1 interacts with ICAM-3 while in a low-affinity conformation ^12^. Likewise, LFA-3 interacts with CD2 with suitable low affinity ^13^, although the glycocalyces are likely to hinder their interaction sterically ^14^. These contacts allow the transient interaction of the TCR with peptide-loaded MHC. If such interaction bears enough affinity, it overcomes the repulsive forces between the glycocalyces; if not, repulsion dominates and the unproductive contact between the mismatched T cell and APC is resolved.

### TCR ligation and initial signaling

Successful interaction of the TCR/CD3 complex with peptide-loaded MHC initiates signaling. It is important to point out that very few TCR-MHC interactions are sufficient to trigger T cell activation ^15^. Recent reviews have described the current viewpoints on TCR/CD3 signaling ^16, 17^. Here, we will focus on several aspects of TCR binding and initial signaling that are specific to IS formation and shape the rest of the process.

Productive TCR engagement promotes its immobilization and clustering in the contact area ^18^. This is mediated in part by its interaction with the MHC on the APC, which restricts the possible lateral movement of the TCR to the interacting portion of the plasma membrane of the T cell with the APC. However, the TCR/CD3 complex appears more immobile and clustered than predicted by a model of free diffusion in a semi-planar layer ^8^, suggesting additional mechanisms of immobilization and aggregation. A crucial mechanism is the association of the TCR/CD3 complex with the actin cortex ^19, 20^. A recent study has shown that ligated TCR/CD3 molecules modify the flow of actin underneath them, indicating binding-dependent interactions between the TCR and cortical actin ^21^, which are essential for sustained TCR-dependent signaling ^22^. Such interaction is not direct but relies on the recruitment of actin-binding adaptors, e.g. Nck ^23^.

**Figure 1.**
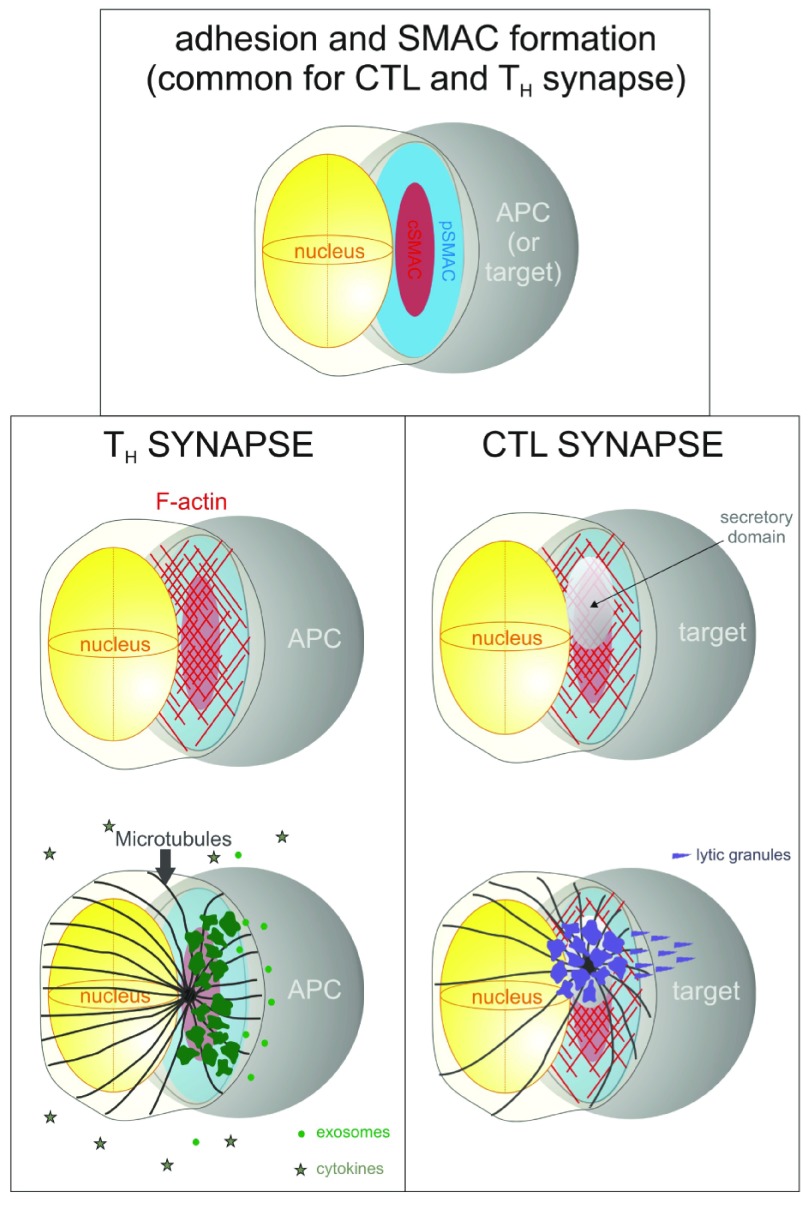
Key events during the formation of the immune synapse. *Top*, diagram represents the adhesion of the T cell (left) to the antigen-presenting cell (APC) (right), and the early formation of discrete domains, central supramolecular activation cluster (cSMAC) (red) containing the T cell receptor (TCR)/CD3 complex and signaling proteins, and the peripheral SMAC (pSMAC) (blue) displaying integrins and their adaptor proteins. *Bottom left column*, events in the T helper (TH) formation of a synapse with a professional APC, including F-actin accumulation ( *top*, in red) and juxtaposition of the secretory apparatus (green) and the microtubule-directing centrosome ( *bottom*, in black), resulting in the polarized secretion of exosomes (bright-green spheres) and the non-polarized secretion of cytokines (stars). *Bottom right column*, events in the CD8+ T (CTL) synapse, including F-actin accumulation and the formation of a secretory domain with weak actin presence ( *top*) and the juxtaposition of the secretory apparatus (purple) and the microtubule-directing centrosome ( *bottom*, in black), resulting in the highly polarized secretion of lytic particles that kill the target cell.

Another important topic is cluster size. There is evidence of small (nanosized) TCR clusters even before their interaction with the MHC. These nanoclusters are continually generated throughout the plasma membrane of the T cell ^24^ and migrate and coalesce at the center of the contact to form micron-scale structures, termed central Supra molecular Activation Clusters (cSMACs) ( Figure 1, top) ^25^, which concentrate signaling components (reviewed in 26) as well as molecules involved in co-stimulation, e.g. CD28 ^27^. The mechanism of coalescence is also unclear, but it also depends on actin and TCR ligation ^28^. Possible explanations involve increases in homotypic TCR lateral affinity, actin coalescence that would “drag” the TCR nanoclusters together, or changes to the size/position of the membrane nanoclusters based on alterations to the regional composition of the plasma membrane. The principles of spatiotemporal assembly of such structures remain unclear, mainly because of differences depending on the type of T cell and APC. In general, T cells that bear a higher basal activation state (e.g. leukemic T cells or memory T cells) form large clusters more readily than resting, naïve T cells. In the latter, TCR/CD3 clusters often remain small and sparse along the contact area between the T cell and the APC ^29, 30^. The difference could pertain to the expression of additional components in activated cells that promote, or facilitate, TCR/CD3 clustering in more pre-activated cells and/or that signals emanating from the TCR/CD3 are more intense in pre-activated cells owing to a higher activation baseline.

### Adhesive interactions

TCR-dependent inside-out signals trigger the conformational extension of integrin LFA-1, enabling its interaction with APC-expressed ICAM-1 (reviewed in 31). This process is similar to the inside-out signaling that activates integrins during extravasation ^32^, and it results in stable adhesion between the APC and the T cell.

**Figure 2.**
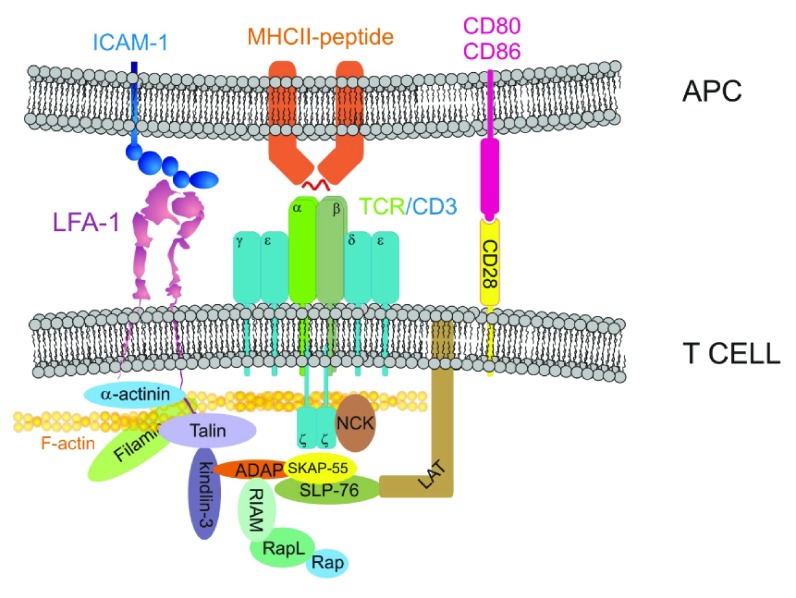
T cell receptor-dependent transactivation of LFA-1. Diagram depicts the major interactions involved in actin-dependent T cell receptor (TCR) and integrin immobilization at the immune synapse (IS), including the signaling modules involved in LFA-1 transactivation. The diagram focuses on the role of SLP-76/ADAP/SKAP-55 in recruiting kindlin-3 and RIAM in proximity to integrin, and the role of Rap/RapL/RIAM in promoting talin association with the β chain of the LFA-1 dimer.

TCR signals that mediate LFA-1 trans-activation go through several adaptor circuits, including Rap1-RapL-RIAM and SLP-76/ADAP/SKAP ( Figure 2). Rap1 is a small Ras-like GTPase that is activated by RasGEFs triggered by the TCR, e.g. CalDAG-1. Active Rap1 forms a complex with RapL and RIAM that targets talin to the plasma membrane ^33^, where it promotes the conformational extension of LFA-1 ^34^. SLP-76/ADAP/SKAP-55 bind to the TCR effector LAT, triggering their association to RIAM, thereby participating in the delivery of talin to the integrin ^35^.

Another important molecule for the inside-out activation of LFA-1 via TCR is kindlin-3. Kindlin-3 mutations cause a severe form of immunodeficiency, named Leukocyte Adhesion Deficiency (LAD)-III ^36, 37^. LAD-III T cells do not migrate properly and activate poorly due to impaired adhesion mediated by LFA-1 ^38^. There are two possible mechanisms to explain the role of kindlin-3 in LFA-1 transactivation. One mechanism postulates that kindlin-3 triggers inside-out activation of LFA-1 by binding directly to the β chain cytoplasmic domain. The other mechanism suggests that kindlin-3 could facilitate the binding of talin, or its effect on the conformational extension of LFA-1 (reviewed in 39). Recruitment of kindlin-3 to LFA-1 is likely mediated by its interaction with ADAP, as in platelet integrin α _IIB_β _3_^40^ ( Figure 2).

LFA-1 is the predominant integrin that mediates the interaction of T _H_ cells with APC. It is also important for the formation of IS between CTL and target cells. However, it is unlikely that every target cell expresses ICAM-1, thus additional integrins may be implicated in the formation of IS. Prior studies have described possible roles for VLA-4 (α _4_β _1_) and VLA-5 (α _5_β _1_) in the IS (reviewed in 41), but their ligands as well as their redundant/unique functions with respect to LFA-1 remain unclear. Spatially, integrins localize throughout the contact area of the T cell and the APC. In activated cells (e.g. super-antigen-triggered clonal leukemic T cells), integrins localize in the outer edge of the contact zone, defining a peripheral SMAC (pSMAC) ( Figure 1, top).

### Actin reorganization at the IS

Outside-in signals stemming from the TCR and integrins promote actin polymerization and clustering at the T cell:APC interface ( Figure 1). As discussed above, actin accumulation is fundamental for the clustering of the TCR and the integrins, forming a positive feedback loop. TCR/CD3 and integrins trigger actin polymerization through several pathways. A major pathway of TCR-mediated actin polymerization depends on the small GTPase Rac1. The TCR activates several Rac GEFs, including Vav1 ^42^ and Tiam1 ^43^. Rac promotes branched actin accumulation by activating a multi-molecular complex that includes WAVE (Scar), HSP300, ABL2, SRA1, and NAP1. This complex associates with the Arp2/3 complex, triggering actin polymerization, as reviewed elsewhere ^44^. Wiskott-Aldrich syndrome protein (WASP) is a protein related to WAVE that also induces Arp2/3-dependent actin polymerization downstream of the TCR, but it is activated by the small GTPase Cdc42 ^45^.

The contribution of other mechanisms of actin polymerization to the congregation of actin at the contact area with the APC is less clear. During the first steps of the formation of the IS, molecular regulators of actin assembly, e.g. ADF/cofilin, are involved in the dynamic reorganization and accumulation of actin at the contact region. For example, depletion of ADF/cofilin function in T cells enhances the accumulation of actin at the IS ^46^. Formins, e.g. mDia, are barbed end nucleators that bind to the uncapped actin filament through one domain and to G-actin-loaded profilin through another, thereby catalyzing G-actin transfer from profilin to the barbed end. mDia-deficient T cells activate and migrate deficiently ^47^. Finally, the Arp2/3 complex, which nucleates dendritic actin polymerization at the lamellipodium of migrating cells ^48^, also participates in the formation of actin lamellae at the IS, although differently shaped actin can accumulate at the IS in the absence of the Arp2/3 complex, in a formin-dependent manner ^49^.

Actin accumulation is also regulated by the function of actin-binding proteins involved in its cross-linking. For example, α-actinin and filamin accumulate at the IS and are required for proper T cell activation in response to antigen-loaded MHC ^50, 51^. It is important to note that these two actin cross-linkers also bind directly to the cytoplasmic tail of β integrins ^52, 53^ ( Figure 2), hence they play a dual function facilitating actin and integrin accumulation at the synapse. Other cross-linkers, e.g. non-muscle myosin II (NMII), are also involved in the formation of efficient synapses. However, the role of NMII in IS formation is controversial. Some studies have shown that NMII affects TCR clustering into the cSMAC ^54, 55^, likely due to impaired actin-dependent flux of the TCR towards the contact area ^56^, but other studies suggest a minimal involvement of this molecule in the formation of the IS ^57, 58^. The differences between these studies likely reside in the type of T cell and APC used. NMII may play an additional role by regulating the mechanics of the contact interface of the T cell and the APC. In this regard, changes to the rigidity of the APC surface (and NMII inhibition) affect T cell activation ^59^, indicating that the mechanics of the interfacing surfaces also play a role in the process.

### Polarization of the secretory apparatus and the centrosome

TCR and integrin signaling promotes a dramatic redistribution of cellular components in the T cell, most notably the redistribution of the secretory apparatus (centrosome and Golgi, reviewed recently in 60) and machinery involved in the generation of extracellular vesicles ^61^ towards the contact area with the APC ( Figure 1, both columns). A major difference with the neuronal synapse is that the secretory apparatus of the APC does not polarize towards the post-synaptic cell (the T cell). This is a crucial event during this process that is often used as a marker of IS maturation. It depends on the activation of microtubule motors, e.g. dynein, which “reel in” the centrosome and the associated secretory elements towards the signaling area. This process has been reviewed in detail elsewhere ^62– 64^. In IS formed between CTL and target cells, this polarization ensures the rapid and specific lysis of the target cell ( Figure 1, bottom right column, and next two sections). A major argument to explain the polarization of the secretory apparatus in T _H_ cells has emerged recently with the discovery of the unidirectional transmission of microRNA-containing exosomes from the T _H_ cell to the APC ^65^ ( Figure 1, bottom left column), which could influence the activation state of the APC, inducing functional activation or anergy of the APC depending on the microRNAs contained in the exosomes.

### Formation of a secretory domain in the CTL synapse

Actin accumulation at the IS facilitates the initial activation of the T cell by immobilizing receptors involved in the contact with the APC and sustaining localized signaling. However, it also constitutes a steric hindrance for polarized secretion. In the early 2000s, Griffiths’ group described the clearance of a part of central actin in maturing cytotoxic IS ( Figure 1, right column). Such a zone, containing less actin than its surroundings, coincided with the localization of intracellular granzyme ^66^, suggesting that the region of actin clearance acted as a gate that enabled efficient secretion towards the target cell. However, recent studies have indicated that very small openings in the cortical actin may be sufficient for efficient vesicle delivery ^67, 68^. The mechanism of actin clearance at the cytotoxic synapse remains unclear. A recent study indicates that coronin 1A is a key mediator of actin remodeling and clearance at the contact area to form the secretory domain ^69^. The contribution of other actin mediators of depolymerization, e.g. cofilin, has been suggested but not directly demonstrated ^70^. This scenario implicates that the depolymerization signal stems from receptors localized at the CTL side of the IS. An intriguing possibility, untested yet, is that secretory granules directly depolymerize actin at the IS by carrying actin remodeling factors in their surface.

### Target cell killing/T cell activation

In the case of pre-primed CTL-contacting target cells bearing antigen-loaded MHC-I, the subsequent steps of this process involve the secretion of granzyme- and perforin-loaded vesicles to kill the target cell ( Figure 1, bottom right column). This has been reviewed in detail elsewhere ^71– 73^. Before that, naïve CTLs undergo priming (i.e. expression of lytic enzymes and their load into the secretory apparatus) at the secondary lymphoid organs (SLOs) when they enter into contact with mature DCs bearing suitable antigens associated with MHC-I. Direct priming occurs only when a) the pathogen infects and activates DCs directly and b) the pathogen-infected cell (or tumor cell) migrates directly into the SLO. Importantly, the establishment of IS between naïve CTLs and immature DCs leads to cross-tolerance, i.e. the inability of the CTL to activate properly ^74^. This is likely an important mechanism of induction of tolerance involved in tumor evasion.

On the other hand, T _H_–APC contacts trigger a transcriptional program that results in the activation of the T _H_, including expression of activation markers, e.g. CD69 and CD25, and cytokine secretion, e.g. IFN-γ and IL-2 ( Figure 1, bottom left column). The main function of these cytokines is to create an activating microenvironment for other immune cells in a paracrine manner. At the site of infection, these cytokines activate other effector cells, particularly macrophages involved in pathogen clearance, CTLs, and NK cells.

Additional molecules induced by the establishment of IS include mediators of cell proliferation downstream of NF-AT, AP1, and NF-kB (reviewed in 75) as well as receptors implicated in the migration of the activated cell to the inflammatory site, e.g. CCR5 ^76^.

### IS termination

The specific signals that promote termination of the IS are unclear. In the case of IS of CTL with target cells, a clear candidate to promote termination of the contact is the flip-flop of the plasma membrane of the target cell due to the effect of the lytic enzymes secreted by the CTL. In such a mechanism, the CTL would recognize phosphatidylserine, annexin V, or other components of the inner leaflet of the plasma membrane of the target cell. In the case of naïve CTL or T _H_ cells, the mechanism is less clear but likely involves the exhaustion of the TCR recycling process over extended periods of stimulation ^77^. Importantly, signaling molecules involved in the formation and function of the IS, e.g. PKCtheta, are also involved in synapse breakdown, constituting a possible mechanism of early remodeling of the IS ^78^.

## Concluding remarks: towards the application of manipulating the IS in biomedicine

In recent years, the need for new therapies against multidrug-resistant tumors and the secondary effects of current therapies, e.g. chemotherapy, have led to the study and the development of better "targeted" therapies with less deleterious side effects for patients. Therefore, enhancing the ability of the immune system to detect and remove pathological cells through recognition of tumor or different expression patterns of the target cells is a crucial step to develop better therapies. Another important issue is to counteract the evasive mechanisms developed by pathogens and tumor cells.

**Figure 3.**
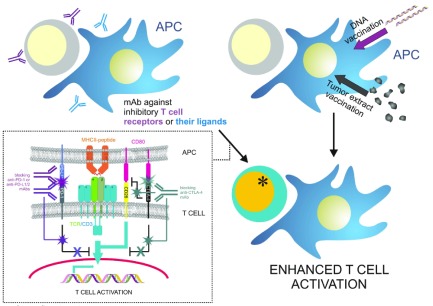
Therapy-based enhancement of immune synapse formation between T cells and tumor antigen-presenting dendritic cells. *Top left*, poorly responding T cells are treated with antibodies that block inhibitory molecules such as CTLA-4 and PD-1, or inhibitory ligands of the latter, e.g. PD-L1/2. *Bottom left inlay*, representation of the effect of anti-CTLA-4 blockade, which blocks inhibitory signals emanating from CTLA-4 that counteract TCR/CD3-dependent signals and also releases CD80 to co-stimulate via interaction with CD28; also depicted is the effect of anti-PD-1 or anti-PD-L1/2 monoclonal antibodies (mAbs), which prevent their interaction and the generation of inhibitory signals. *Top right*, direct vaccination of dendritic cells with tumor DNA or autologous or allogeneic tumor extracts. *Bottom right*, either treatment should enhance T cell response against tumor antigens.

One approach aimed at improving the immune response against tumor cells consists of autologous or allogeneic tumor vaccination ( Figure 3, top right). These approaches are aimed at generating strong CTL responses against tumor cells based on their specific molecular makeup. The underlying mechanism consists of vaccine-mediated CTL priming by vaccine-stimulated APC (mainly DCs), which would then home to the tumor and rapidly form an IS with the tumor cells, killing them. Several trials based on this approach are reviewed here ^79^. Another possibility is the genetic immunization of patients (DNA vaccination) through DCs. The major limiting factor is the need for safe and specific carriers. An attractive possibility is the use of *in vivo* DC-targeting liposomal DNA vaccine carriers ^80^.

Approaches aimed at suppressing the effects of the evasive maneuvers of tumor cells have also been tested in recent years ( Figure 3, top left). For example, tumor cells are believed to promote the expression of CTLA-4, which is a molecule expressed by T cells that competes with CD28 for the co-stimulatory molecule CD80 (B7.1), thereby suppressing T cell activation. The US Food and Drug Administration (FDA) and the European Medicines Agency (EMA) have approved the use of a humanized monoclonal antibody against CTLA-4 for the treatment of late-stage melanoma ^81^. Similar approaches have been developed for PD-1, which is another inhibitory receptor that suppresses T cell responses independent of CD28 but dependent of its ligands PD-L1 and PD-L2, which are abundantly expressed by several types of tumor cells ^82^. A number of antibodies against PD-1 and PD-L1/2 are being developed by big pharmaceutical companies aiming to find different anti-tumor therapies ^83– 85^. At a molecular level, CTLA-4 binding to CD28 disrupts TCR clustering, effectively destabilizing the IS ^86^. Likewise, PD-1 accumulation at the IS recruits protein phosphatases, such as SHP-2, that quench the stimulating signals emanating from the synapse ^87^.

Clearly, these studies and novel forms of treatment are of outstanding importance in the development of new treatments for the more aggressive and less-tractable types of cancer and are likely the beginning of a new era of molecular treatment of cancer.

## References

[1] Norcross MA: A synaptic basis for T-lymphocyte activation. *Ann Immunol (Paris).* 1984;135D(2):113–34. 10.1016/S0769-2625(84)81105-8 6151375 PMC2551763

[2] Paul WE Seder RA: Lymphocyte responses and cytokines. *Cell.* 1994;76(2):241–51. 10.1016/0092-8674(94)90332-8 7904900

[3] Monks CR Freiberg BA Kupfer H: Three-dimensional segregation of supramolecular activation clusters in T cells. *Nature.* 1998;395(6697):82–6. 10.1038/25764 9738502

[4] Dustin ML Olszowy MW Holdorf AD: A novel adaptor protein orchestrates receptor patterning and cytoskeletal polarity in T-cell contacts. *Cell.* 1998;94(5):667–77. 10.1016/S0092-8674(00)81608-6 9741631

[5] Dustin ML: Signaling at neuro/immune synapses. *J Clin Invest.* 2012;122(4):1149–55. 10.1172/JCI58705 22466656 PMC3314453

[6] Miller MJ Wei SH Parker I: Two-photon imaging of lymphocyte motility and antigen response in intact lymph node. *Science.* 2002;296(5574):1869–73. 10.1126/science.1070051 12016203

[7] Stoll S Delon J Brotz TM: Dynamic imaging of T cell-dendritic cell interactions in lymph nodes. *Science.* 2002;296(5574):1873–6. 10.1126/science.1071065 12052961

[8] Dustin ML Depoil D: New insights into the T cell synapse from single molecule techniques. *Nat Rev Immunol.* 2011;11(10):672–84. 10.1038/nri3066 21904389 PMC3889200

[9] Grigorian A Torossian S Demetriou M: T-cell growth, cell surface organization, and the galectin-glycoprotein lattice. *Immunol Rev.* 2009;230(1):232–46. 10.1111/j.1600-065X.2009.00796.x 19594640 PMC3059806

[10] Molon B Gri G Bettella M: T cell costimulation by chemokine receptors. *Nat Immunol.* 2005;6(5):465–71. 10.1038/ni1191 15821738

[11] Montoya MC Sancho D Bonello G: Role of ICAM-3 in the initial interaction of T lymphocytes and APCs. *Nat Immunol.* 2002;3(2):159–68. 10.1038/ni753 11812993

[12] de Fougerolles AR Qin X Springer TA: Characterization of the function of intercellular adhesion molecule (ICAM)-3 and comparison with ICAM-1 and ICAM-2 in immune responses. *J Exp Med.* 1994;179(2):619–29. 10.1084/jem.179.2.619 7905020 PMC2191386

[13] van der Merwe PA Barclay AN Mason DW: Human cell-adhesion molecule CD2 binds CD58 (LFA-3) with a very low affinity and an extremely fast dissociation rate but does not bind CD48 or CD59. *Biochemistry.* 1994;33(33):10149–60. 10.1021/bi00199a043 7520278

[14] Dustin ML Ferguson LM Chan PY: Visualization of CD2 interaction with LFA-3 and determination of the two-dimensional dissociation constant for adhesion receptors in a contact area. *J Cell Biol.* 1996;132(3):465–74. 10.1083/jcb.132.3.465 8636222 PMC2120727

[15] Irvine DJ Purbhoo MA Krogsgaard M: Direct observation of ligand recognition by T cells. *Nature.* 2002;419(6909):845–9. 10.1038/nature01076 12397360

[16] Chakraborty AK Weiss A: Insights into the initiation of TCR signaling. *Nat Immunol.* 2014;15(9):798–807. 10.1038/ni.2940 25137454 PMC5226627

[17] Malissen B Grégoire C Malissen M: Integrative biology of T cell activation. *Nat Immunol.* 2014;15(9):790–7. 10.1038/ni.2959 25137453

[18] Grakoui A Bromley SK Sumen C: The immunological synapse: a molecular machine controlling T cell activation. *Science.* 1999;285(5425):221–7. 10.1126/science.285.5425.221 10398592

[19] Beemiller P Krummel MF: Regulation of T-cell receptor signaling by the actin cytoskeleton and poroelastic cytoplasm. *Immunol Rev.* 2013;256(1):148–59. 10.1111/imr.12120 24117819 PMC3831008

[20] Billadeau DD Nolz JC Gomez TS: Regulation of T-cell activation by the cytoskeleton. *Nat Rev Immunol.* 2007;7(2):131–43. 10.1038/nri2021 17259969

[21] Smoligovets AA Smith AW Wu HJ: Characterization of dynamic actin associations with T-cell receptor microclusters in primary T cells. *J Cell Sci.* 2012;125(Pt 3):735–42. 10.1242/jcs.092825 22389407 PMC3367833

[22] Babich A Li S O'Connor RS: F-actin polymerization and retrograde flow drive sustained PLCγ1 signaling during T cell activation. *J Cell Biol.* 2012;197(6):775–87. 10.1083/jcb.201201018 22665519 PMC3373411

[23] Gil D Schamel WW Montoya M: Recruitment of Nck by CD3 epsilon reveals a ligand-induced conformational change essential for T cell receptor signaling and synapse formation. *Cell.* 2002;109(7):901–12. 10.1016/S0092-8674(02)00799-7 12110186

[24] Yokosuka T Sakata-Sogawa K Kobayashi W: Newly generated T cell receptor microclusters initiate and sustain T cell activation by recruitment of Zap70 and SLP-76. *Nat Immunol.* 2005;6(12):1253–62. 10.1038/ni1272 16273097

[25] Saito T Yokosuka T Hashimoto-Tane A: Dynamic regulation of T cell activation and co-stimulation through TCR-microclusters. *FEBS Lett.* 2010;584(24):4865–71. 10.1016/j.febslet.2010.11.036 21110974

[26] Dustin ML Groves JT: Receptor signaling clusters in the immune synapse. *Annu Rev Biophys.* 2012;41:543–56. 10.1146/annurev-biophys-042910-155238 22404679 PMC4000727

[27] Pentcheva-Hoang T Egen JG Wojnoonski K: B7-1 and B7-2 selectively recruit CTLA-4 and CD28 to the immunological synapse. *Immunity.* 2004;21(3):401–13. 10.1016/j.immuni.2004.06.017 15357951

[28] Bunnell SC Kapoor V Trible RP: Dynamic actin polymerization drives T cell receptor-induced spreading: a role for the signal transduction adaptor LAT. *Immunity.* 2001;14(3):315–29. 10.1016/S1074-7613(01)00112-1 11290340

[29] Brossard C Feuillet V Schmitt A: Multifocal structure of the T cell - dendritic cell synapse. *Eur J Immunol.* 2005;35(6):1741–53. 10.1002/eji.200425857 15909310

[30] Thauland TJ Parker DC: Diversity in immunological synapse structure. *Immunology.* 2010;131(4):466–72. 10.1111/j.1365-2567.2010.03366.x 21039474 PMC2999798

[31] Shattil SJ Kim C Ginsberg MH: The final steps of integrin activation: the end game. *Nat Rev Mol Cell Biol.* 2010;11(4):288–300. 10.1038/nrm2871 20308986 PMC3929966

[32] Peled A Kollet O Ponomaryov T: The chemokine SDF-1 activates the integrins LFA-1, VLA-4, and VLA-5 on immature human CD34(+) cells: role in transendothelial/stromal migration and engraftment of NOD/SCID mice. *Blood.* 2000;95(11):3289–96. 10828007

[33] Lee HS Lim CJ Puzon-McLaughlin W: RIAM activates integrins by linking talin to ras GTPase membrane-targeting sequences. *J Biol Chem.* 2009;284(8):5119–27. 10.1074/jbc.M807117200 19098287 PMC2643525

[34] Hogg N Patzak I Willenbrock F: The insider's guide to leukocyte integrin signalling and function. *Nat Rev Immunol.* 2011;11(6):416–26. 10.1038/nri2986 21597477

[35] Ménasché G Kliche S Chen EJ: RIAM links the ADAP/SKAP-55 signaling module to Rap1, facilitating T-cell-receptor-mediated integrin activation. *Mol Cell Biol.* 2007;27(11):4070–81. 10.1128/MCB.02011-06 17403904 PMC1900018

[36] Malinin NL Zhang L Choi J: A point mutation in *KINDLIN3* ablates activation of three integrin subfamilies in humans. *Nat Med.* 2009;15(3):313–8. 10.1038/nm.1917 19234460 PMC2857384

[37] Svensson L Howarth K McDowall A: Leukocyte adhesion deficiency-III is caused by mutations in *KINDLIN3* affecting integrin activation. *Nat Med.* 2009;15(3):306–12. 10.1038/nm.1931 19234463 PMC2680140

[38] Manevich-Mendelson E Feigelson SW Pasvolsky R: Loss of Kindlin-3 in LAD-III eliminates LFA-1 but not VLA-4 adhesiveness developed under shear flow conditions. *Blood.* 2009;114(11):2344–53. 10.1182/blood-2009-04-218636 19617577

[39] Fagerholm SC Lek HS Morrison VL: Kindlin-3 in the immune system. *Am J Clin Exp Immunol.* 2014;3(1):37–42. 24660120 PMC3960760

[40] Kasirer-Friede A Kang J Kahner B: ADAP interactions with talin and kindlin promote platelet integrin αIIbβ3 activation and stable fibrinogen binding. *Blood.* 2014;123(20):3156–65. 10.1182/blood-2013-08-520627 24523237 PMC4023421

[41] Kinashi T: Intracellular signalling controlling integrin activation in lymphocytes. *Nat Rev Immunol.* 2005;5(7):546–59. 10.1038/nri1646 15965491

[42] Saveliev A Vanes L Ksionda O: Function of the nucleotide exchange activity of vav1 in T cell development and activation. *Sci Signal.* 2009;2(101):ra83. 10.1126/scisignal.2000420 20009105 PMC3434450

[43] Grönholm M Jahan F Marchesan S: TCR-induced activation of LFA-1 involves signaling through Tiam1. *J Immunol.* 2011;187(7):3613–9. 10.4049/jimmunol.1100704 21876037

[44] Rotty JD Wu C Bear JE: New insights into the regulation and cellular functions of the ARP2/3 complex. *Nat Rev Mol Cell Biol.* 2013;14(1):7–12. 10.1038/nrm3492 23212475

[45] Matalon O Reicher B Barda-Saad M: Wiskott-Aldrich syndrome protein--dynamic regulation of actin homeostasis: from activation through function and signal termination in T lymphocytes. *Immunol Rev.* 2013;256(1):10–29. 10.1111/imr.12112 24117810

[46] Kim J Shapiro MJ Bamidele AO: Coactosin-like 1 antagonizes cofilin to promote lamellipodial protrusion at the immune synapse. *PLoS One.* 2014;9(1):e85090. 10.1371/journal.pone.0085090 24454796 PMC3890291

[47] Sakata D Taniguchi H Yasuda S: Impaired T lymphocyte trafficking in mice deficient in an actin-nucleating protein, mDia1. *J Exp Med.* 2007;204(9):2031–8. 10.1084/jem.20062647 17682067 PMC2118705

[48] Pollard TD Borisy GG: Cellular motility driven by assembly and disassembly of actin filaments. *Cell.* 2003;112(4):453–65. 10.1016/S0092-8674(03)00120-X 12600310

[49] Gomez TS Kumar K Medeiros RB: Formins regulate the actin-related protein 2/3 complex-independent polarization of the centrosome to the immunological synapse. *Immunity.* 2007;26(2):177–90. 10.1016/j.immuni.2007.01.008 17306570 PMC2836258

[50] Gordón-Alonso M Sala-Valdés M Rocha-Perugini V: EWI-2 association with α-actinin regulates T cell immune synapses and HIV viral infection. *J Immunol.* 2012;189(2):689–700. 10.4049/jimmunol.1103708 22689882

[51] Hayashi K Altman A: Filamin A is required for T cell activation mediated by protein kinase C-theta. *J Immunol.* 2006;177(3):1721–8. 10.4049/jimmunol.177.3.1721 16849481

[52] Loo DT Kanner SB Aruffo A: Filamin binds to the cytoplasmic domain of the beta1-integrin. Identification of amino acids responsible for this interaction. *J Biol Chem.* 1998;273(36):23304–12. 10.1074/jbc.273.36.23304 9722563

[53] Otey CA Vasquez GB Burridge K: Mapping of the alpha-actinin binding site within the beta 1 integrin cytoplasmic domain. *J Biol Chem.* 1993;268(28):21193–7. 7691808

[54] Ilani T Vasiliver-Shamis G Vardhana S: T cell antigen receptor signaling and immunological synapse stability require myosin IIA. *Nat Immunol.* 2009;10(5):531–9. 10.1038/ni.1723 19349987 PMC2719775

[55] Kumari S Vardhana S Cammer M: T Lymphocyte Myosin IIA is Required for Maturation of the Immunological Synapse. *Front Immunol.* 2012;3:230. 10.3389/fimmu.2012.00230 22912631 PMC3421155

[56] Yu Y Fay NC Smoligovets AA: Myosin IIA modulates T cell receptor transport and CasL phosphorylation during early immunological synapse formation. *PLoS One.* 2012;7(2):e30704. 10.1371/journal.pone.0030704 22347397 PMC3275606

[57] Jacobelli J Chmura SA Buxton DB: A single class II myosin modulates T cell motility and stopping, but not synapse formation. *Nat Immunol.* 2004;5(5):531–8. 10.1038/ni1065 15064761

[58] Beemiller P Jacobelli J Krummel MF: Integration of the movement of signaling microclusters with cellular motility in immunological synapses. *Nat Immunol.* 2012;13(8):787–95. 10.1038/ni.2364 22751140 PMC3902181

[59] Judokusumo E Tabdanov E Kumari S: Mechanosensing in T lymphocyte activation. *Biophys J.* 2012;102(2):L5–7. 10.1016/j.bpj.2011.12.011 22339876 PMC3260692

[60] Stinchcombe JC Griffiths GM: Communication, the centrosome and the immunological synapse. *Philos Trans R Soc Lond B Biol Sci.* 2014;369(1650): pii: 20130463. 10.1098/rstb.2013.0463 25047617 PMC4113107

[61] Choudhuri K Llodrá J Roth EW: Polarized release of T-cell-receptor-enriched microvesicles at the immunological synapse. *Nature.* 2014;507(7490):118–23. 10.1038/nature12951 24487619 PMC3949170

[62] Martín-Cófreces NB Baixauli F Sánchez-Madrid F: Immune synapse: conductor of orchestrated organelle movement. *Trends Cell Biol.* 2014;24(1):61–72. 10.1016/j.tcb.2013.09.005 24119664 PMC4347664

[63] Soares H Lasserre R Alcover A: Orchestrating cytoskeleton and intracellular vesicle traffic to build functional immunological synapses. *Immunol Rev.* 2013;256(1):118–32. 10.1111/imr.12110 24117817

[64] Yadav S Linstedt AD: Golgi positioning. *Cold Spring Harb Perspect Biol.* 2011;3(5): pii: a005322. 10.1101/cshperspect.a005322 21504874 PMC3101843

[65] Mittelbrunn M Gutiérrez-Vázquez C Villarroya-Beltri C: Unidirectional transfer of microRNA-loaded exosomes from T cells to antigen-presenting cells. *Nat Commun.* 2011;2: 282. 10.1038/ncomms1285 21505438 PMC3104548

[66] Stinchcombe JC Bossi G Booth S: The immunological synapse of CTL contains a secretory domain and membrane bridges. *Immunity.* 2001;15(5):751–61. 10.1016/S1074-7613(01)00234-5 11728337

[67] Brown AC Oddos S Dobbie IM: Remodelling of cortical actin where lytic granules dock at natural killer cell immune synapses revealed by super-resolution microscopy. *PLoS Biol.* 2011;9(9):e1001152. 10.1371/journal.pbio.1001152 21931537 PMC3172219

[68] Rak GD Mace EM Banerjee PP: Natural killer cell lytic granule secretion occurs through a pervasive actin network at the immune synapse. *PLoS Biol.* 2011;9(9):e1001151. 10.1371/journal.pbio.1001151 21931536 PMC3172191

[69] Mace EM Orange JS: Lytic immune synapse function requires filamentous actin deconstruction by Coronin 1A. *Proc Natl Acad Sci U S A.* 2014;111(18):6708–13. 10.1073/pnas.1314975111 24760828 PMC4020046

[70] Mace EM Dongre P Hsu HT: Cell biological steps and checkpoints in accessing NK cell cytotoxicity. *Immunol Cell Biol.* 2014;92(3):245–55. 10.1038/icb.2013.96 24445602 PMC3960583

[71] Susanto O Trapani JA Brasacchio D: Controversies in granzyme biology. *Tissue Antigens.* 2012;80(6):477–87. 10.1111/tan.12014 23137319

[72] Williams MA Bevan MJ: Effector and memory CTL differentiation. *Annu Rev Immunol.* 2007;25:171–92. 10.1146/annurev.immunol.25.022106.141548 17129182

[73] Stinchcombe JC Griffiths GM: Secretory mechanisms in cell-mediated cytotoxicity. *Annu Rev Cell Dev Biol.* 2007;23:495–517. 10.1146/annurev.cellbio.23.090506.123521 17506701

[74] Melief CJ: Mini-review: Regulation of cytotoxic T lymphocyte responses by dendritic cells: peaceful coexistence of cross-priming and direct priming? *Eur J Immunol.* 2003;33(10):2645–54. 10.1002/eji.200324341 14515248

[75] Padhan K Varma R: Immunological synapse: a multi-protein signalling cellular apparatus for controlling gene expression. *Immunology.* 2010;129(3):322–8. 10.1111/j.1365-2567.2009.03241.x 20409153 PMC2826677

[76] Ebert LM McColl SR: Up-regulation of CCR5 and CCR6 on distinct subpopulations of antigen-activated CD4 ^+^ T lymphocytes. *J Immunol.* 2002;168(1):65–72. 10.4049/jimmunol.168.1.65 11751947

[77] Lasserre R Cuche C Blecher-Gonen R: Release of serine/threonine-phosphorylated adaptors from signaling microclusters down-regulates T cell activation. *J Cell Biol.* 2011;195(5):839–53. 10.1083/jcb.201103105 22105350 PMC3257567

[78] Sims TN Soos TJ Xenias HS: Opposing effects of PKCtheta and WASp on symmetry breaking and relocation of the immunological synapse. *Cell.* 2007;129(4):773–85. 10.1016/j.cell.2007.03.037 17512410

[79] Srivatsan S Patel JM Bozeman EN: Allogeneic tumor cell vaccines: the promise and limitations in clinical trials. *Hum Vaccin Immunother.* 2014;10(1):52–63. 10.4161/hv.26568 24064957 PMC4181031

[80] Garu A Moku G Gulla SK: Genetic Immunization With *In Vivo* Dendritic Cell-targeting Liposomal DNA Vaccine Carrier Induces Long-lasting Antitumor Immune Response. *Mol Ther.* 2016;24(2):385–97. 10.1038/mt.2015.215 26666450 PMC4817821

[81] Lipson EJ Drake CG: Ipilimumab: an anti-CTLA-4 antibody for metastatic melanoma. *Clin Cancer Res.* 2011;17(22):6958–62. 10.1158/1078-0432.CCR-11-1595 21900389 PMC3575079

[82] Noh H Hu J Wang X: Immune checkpoint regulator PD-L1 expression on tumor cells by contacting CD11b positive bone marrow derived stromal cells. *Cell Commun Signal.* 2015;13:14. 10.1186/s12964-015-0093-y 25889536 PMC4353689

[83] Topalian SL Hodi FS Brahmer JR: Safety, activity, and immune correlates of anti-PD-1 antibody in cancer. *N Engl J Med.* 2012;366(26):2443–54. 10.1056/NEJMoa1200690 22658127 PMC3544539

[84] Brahmer JR Tykodi SS Chow LQ: Safety and activity of anti-PD-L1 antibody in patients with advanced cancer. *N Engl J Med.* 2012;366(26):2455–65. 10.1056/NEJMoa1200694 22658128 PMC3563263

[85] Ribas A: Tumor immunotherapy directed at PD-1. *N Engl J Med.* 2012;366(26):2517–9. 10.1056/NEJMe1205943 22658126

[86] Jackman RP Balamuth F Bottomly K: CTLA-4 differentially regulates the immunological synapse in CD4 T cell subsets. *J Immunol.* 2007;178(9):5543–51. 10.4049/jimmunol.178.9.5543 17442936

[87] Yokosuka T Takamatsu M Kobayashi-Imanishi W: Programmed cell death 1 forms negative costimulatory microclusters that directly inhibit T cell receptor signaling by recruiting phosphatase SHP2. *J Exp Med.* 2012;209(6):1201–17. 10.1084/jem.20112741 22641383 PMC3371732

